# Colloids Yes or No? - a “Gretchen Question” Answered

**DOI:** 10.3389/fvets.2021.624049

**Published:** 2021-07-02

**Authors:** Katja-Nicole Adamik, Ivayla D. Yozova

**Affiliations:** ^1^Division of Small Animal Emergency and Critical Care, Department of Clinical Veterinary Science, Vetsuisse Faculty, University of Bern, Bern, Switzerland; ^2^School of Veterinary Science, Massey University, Palmerston North, New Zealand

**Keywords:** albumin, dextran, gelatin, HBOC, hydroxyethyl starch, fresh frozen plasma, fluid therapy

## Abstract

Colloid solutions, both natural and synthetic, had been widely accepted as having superior volume expanding effects than crystalloids. Synthetic colloid solutions were previously considered at least as effective as natural colloids, as well as being cheaper and easily available. As a result, synthetic colloids (and HES in particular) were the preferred resuscitation fluid in many countries. In the past decade, several cascading events have called into question their efficacy and revealed their harmful effects. In 2013, the medicines authorities placed substantial restrictions on HES administration in people which has resulted in an overall decrease in their use. Whether natural colloids (such as albumin-containing solutions) should replace synthetic colloids remains inconclusive based on the current evidence. Albumin seems to be safer than synthetic colloids in people, but clear evidence of a positive effect on survival is still lacking. Furthermore, species-specific albumin is not widely available, while xenotransfusions with human serum albumin have known side effects. Veterinary data on the safety and efficacy of synthetic and natural colloids is limited to mostly retrospective evaluations or experimental studies with small numbers of patients (mainly dogs). Large, prospective, randomized, long-term outcome-oriented studies are lacking. This review focuses on advantages and disadvantages of synthetic and natural colloids in veterinary medicine. Adopting human guidelines is weighed against the particularities of our specific patient populations, including the risk–benefit ratio and lack of alternatives available in human medicine.

## Background

A colloid is the collective term for electrolyte solutions containing macromolecules, a portion of which cannot pass freely out of the healthy intravascular space. Hence, they exert a colloid osmotic pressure (COP) and thereby retain or attract water commensurate with the number rather than the size of the colloid molecules on each side of the microvascular barrier (1). Natural colloids include blood products and albumin solutions, such as human serum albumin (HSA) and canine serum albumin (CSA). Synthetic colloids include hydroxyethyl starches (HES), gelatins, and dextrans (2) (Table 1). Synthetic colloids and particularly HES solutions have been preferred as volume expanders over isotonic crystalloids for decades (3). This was based on their presumed “volume-sparing effect.” Indeed, it was accepted that less colloid than isotonic crystalloid is necessary to reach adequate volume expansion in various populations of patients. Furthermore, since synthetic colloids do not readily cross the vascular barrier and produce COP, they were believed to contribute to prevention and reduction of edema formation (2, 4). However, the advantages of synthetic colloids have been questioned in recent years for two main reasons. First, regardless of their proposed short-term benefits, synthetic colloids are now known to be associated with serious long-term adverse effects. Second, the microanatomic and physiological basis of transvascular fluid flux has been revised in light of the more widespread appreciation of the role played by the endothelial surface layer (ESL) (see below). This newer understanding has raised doubts regarding the fundamental validity of the physiological basis for colloid therapy. Nevertheless, according to an international survey from 2016, synthetic colloids and particularly HES were extensively used in small animals (5). Since 2013 pharmacovigilance authorities worldwide have restricted the use of HES in people and colloid usage has therefore shifted to other products (namely, albumin and gelatins) (6). As a result, veterinarians are facing difficulties in procurement and overall reduction in availability of HES. Whether a similar shift toward alternative colloids is possible and safe for veterinary patients is unclear. This review will describe the characteristics and adverse effects of various synthetic and natural colloids, with the aim to provide risk-assessment and evidence-based recommendations for their use.

**Table 1:** Synthetic and natural colloids and their physicochemical characteristics.

**Colloid**	**Colloid Source**	**Concentration**	**Carrier solution**	**Mean MW (kDa)/MS/C2/C6 ratio**	***In vitro* COP (mmHg)**
Plasma	Plasma (dog/cat)	25to 30 g/L	n/a	n/a	17
HSA 5%	Pooled human plasma	50 g/L	Sterile water	~66 kDa	20
HSA 20%, 25%	Pooled human plasma	200–250 g/L	Sterile water	~66 kDa	> 200
CSA^¶^ (5% /16%)	Pooled canine plasma	50/166 g/L	0.9% saline	~60 kDa	n/a
HES, tetrastarch (6% Voluven^*^)	Waxy-maize starch	60 g/L	0.9 saline	130/0.4, 9:1	36
HES, tetrastarch (6% Volulyte^*^)	Waxy-maize starch	60 g/L	Buffered, polyionic	130/0.4, 9:1	36
HES, tetrastarch (Venofundin^†^)	Potato starch	60 g/L	0.9% saline	130/0.42, 6:1	36
HES, tetrastarch (Tetraspan^†^)	Potato starch	60 g/L	Buffered, polyionic	130/0.42, 6:1	36
HES, hetastarch (Hespan^†^)	Waxy-maize starch	60 g/L	0.9% saline	600/0.75	33
HES, hetastarch (Hextend^†^)	Waxy-maize starch	60 g/L	Buffered, polyionic	600/0.75	33
HES, pentastarch (HAES-steril^*^)	Waxy-maize starch	60 g/L	0.9% saline	200/0.5, 5:1	30–35
HES, pentastarch (HyperHAES^*^)	Waxy-maize starch	60 g/L	7.5% saline	200/0.5, 5:1	30–35
Gelatin (Gelofusine 4%^†^)	Bovine collagen	40 g/L	0.9% saline	~30 kDa	33
Gelatin (Gelafundin-Iso^†^)	Bovine collagen	40 g/L	Buffered polyionic	~ 30 kDa	33
10% dextran-40 (Rheomacrodex)	sucrose	100 g/L	0.9% saline	40 kDa	n/a
6% dextran-70 (Macrodex^§^)	sucrose	60 g/L	0.9% saline	70 kDa	62
6% dextran-70 (RescueFlow)	sucrose	60 g/L	7.5% saline	70 kDa	n/a
HBOC (Oxapex)	Bovine hemoglobin	60–70 g/L		65	19

*HBOC, Hemoglobin-based oxygen carrier; HSA, human serum albumin; CSA, canine serum albumin; HES, hydroxyethyl starch; Trade names in brackets, MW, molecular weight; MS, molar substitution; kDa, kilo Dalton; COP, colloid osmotic pressure*.

* *Fresenius Kabi AG, Switzerland*.

† *B. Braun Melsungen AG, Germany*;

§ *Meda, Sweden*;

¶ *HemoSolutions, LLC, Colorado Springs*.

*Names and manufactures for the listed colloids are examples and vary between different countries*.

## Physiology of Colloid Solutions

### Classical vs. Revised Starling Principle

According to the traditional Starling principle, the opposing hydrostatic and colloid osmotic pressures of the intravascular space and the interstitium dictate transvascular fluid fluxes. Thus, administering a fluid that is preferentially retained in the intravascular space and with a COP higher than that of a crystalloid will expand intravascular volume to a greater extent per unit volume administered (1). However, the Starling principle has been revised in recent decades.

The newer “revised” or “extended” Starling principle considers the role of the endothelial glycocalyx (EG) within the ESL as an additional barrier to fluid movement across the vascular wall (7). The EG is a meshwork of membrane-bound complex sugars lining the luminal surface of the endothelium of all mammalian vessels (7). As part of the ESL, it acts as a selective barrier to movement of molecules across the vascular wall, due to its tight entanglement and predominantly negatively charged structure (8). The EG retains albumin and other proteins within the intravascular space. In that way, it is a major determinant of vascular permeability to fluid (9). The EG maintains a relatively low rate of filtration throughout the entire capillary length. At normal hydrostatic pressures, no reabsorption from the interstitium into the intravascular space occurs in non-fenestrated capillaries (10). Contrary to the “classical” Starling principle, the revised Starling principle proposes that the opposing pressures determining fluid extravasation or retention are the capillary hydrostatic pressure, the capillary COP (including that of the EG), and the hydrostatic and colloid osmotic pressure of the sub-glycocalyx area, which is virtually protein free (10). Therefore, in scenarios where the EG is intact, the major determinant of fluid filtration is the capillary hydrostatic pressure. However, in situations where the EG becomes damaged, such as systemic inflammatory states, it can no longer maintain its barrier function and extravasation of both plasma proteins and electrolytes, or administered colloids and crystalloids, is equally likely (11). Furthermore, extravasated colloid solutions increase the interstitial COP, thus retaining more fluid and aggravating interstitial edema (12). Therefore, theoretically, the change in intravascular volume that is achieved from a given dose of crystalloid or colloid depends on the integrity of the individual's EG. It is noteworthy that most of the statements supporting the revised Starling principle are derived from the experimental setting, and the clinical importance of changes in the EG requires determination. Indeed, the clinical significance of some aspects of the revised Starling principle has been called into question in a recent review (13). The interested reader is referred to the article “Advances in the Starling principle and microvascular fluid exchange; consequences and implications for fluid therapy” of this special issue for a more in-depth review on the EG.

### Volume Effects of Colloids

Classically, colloids were believed to exert three to four times the volume effects of crystalloids (14, 15). This has been demonstrated in patients without systemic disease (16), in older studies of people in shock (17, 18), and in experimental hemorrhagic shock models (19–23). Several recent clinical studies, however, failed to corroborate these findings in systemically ill people, suggesting colloid-to-crystalloid relative volume expansion ratios of 1:1–1:1.5 (24). The proposed cause for this discrepancy is the damage to the EG resulting in fluid extravasation of both crystalloids and colloids, and therefore similar volume effects (2). In states of volume depletion, however, colloids seemingly exert a superior volume expanding effect to crystalloids. In people undergoing planned normovolemic hemodilution to a hematocrit of 21%, replacement of the removed blood with 115% volume of 6% HES 130/0.4 restored intravascular volume to 105 ± 4% (25). In a similar setting, replacement of four times the removed blood volume with Ringer's lactate solution led to a volume effect of only 17 ± 10%. In contrast, subsequent infusion of a 20% albumin solution to correct a volume deficit led to a volume effect of 184 ± 63% (16). Several experimental hyper- and normovolemic hemodilution models have shown similar findings, whether by evaluating volume expansion or using resuscitation endpoints (26–28).

Interestingly, these findings contrast with the revised Starling principle, whereby in the transient state of decreased hydrostatic pressure, both crystalloids and colloids should be able to equally restore blood volume (10). The cause for these discrepant findings is probably multifactorial. For instance, they might simply be the result of incomparable study designs or imprecise or different methodology. Indeed, the current techniques for estimating blood volume (such as dilution tracer techniques and the more common measurement of hemodilution) are challenging to perform and may carry inherent errors (12, 13, 29, 30). As a result, comparison between studies is difficult. This further hinders the ability of scientific evidence to translate into meaningful clinical guidelines.

In addition to “superior” volume expansion effects, colloids are proposed to stay “longer in the vessel” than crystalloids. While crystalloids are expected to redistribute over 20–40 min, studies have shown that the colloids persist in the intravascular space for 2–3 h in healthy euvolemic volunteers (31–33). Systemic inflammatory and otherwise “leaky” states are expected to shorten the duration of this effect (34, 35). However, extensive studies on redistribution of colloids are lacking (12). Further, colloids (and especially HES) have been specifically indicated in states of increased capillary permeability to “plug the holes in the endothelium.” The ability of the macromolecules contained in colloids to wedge into endothelial intercellular orifices, a product of systemic inflammation and endothelial dysfunction, has been proclaimed in textbook chapters and review articles (4, 14). This has been extrapolated mostly from experimental models using entire animals (36–42), isolated tissues (43), or endothelial cell lines (44). However, this ability remains to be tested in the clinical setting. Whether colloids reduce or exacerbate fluid extravasation in systemic inflammatory states remains controversial with studies reporting opposing results (34, 45–47). The reader is reminded that evaluating extravasation is challenging in the clinical setting, which might be a plausible explanation for these discrepancies. Another explanation for the “plugging potential” of colloids may lie in their interaction with the EG. Indeed, experimental studies have demonstrated that albumin might exert protective effects on the EG (48–52). In the same series of experiments, HES showed a similar, albeit weaker, protective effect (53). This is proposed to be the result of colloids binding to the glycosaminoglycan side chains of proteoglycans and glycoproteins and stabilizing the scaffolding structure of the EG (54, 55). However, in the majority of those studies, the study fluid was infused before an insult on the EG. Therefore, infusing colloids once the EG damage has occurred might not carry the same protective effect.

### Colloid Osmotic Pressure Optimization

Synthetic colloids have been used as a substitute for albumin in patients with hypoalbuminemia with the rationale that they are capable of restoring plasma COP. Indeed, several veterinary studies in hypoalbuminemic patients have demonstrated that HES stabilizes (56) or increases plasma COP (57–59), with one study demonstrating a subjective decrease in peripheral edema when using high molecular weight HES (hetastarch) (60). However, the decrease in edema was not correlated with the dose administered and a drop of COP was noted after subsequent HES administrations. In another study where COP increased after administration of HES, it returned to baseline within 12 h of administration (58).

Administering synthetic colloids as a constant rate infusion (CRI) seems to be unique to veterinary medicine. Veterinarians often use synthetic colloids as CRIs for patients with hypoalbuminemia and edema (5). Non–peer-reviewed conference proceedings (61, 62) and review articles (2) recommend doses of 1–2 ml/kg/h or the maximal recommended daily dose divided by 24 h to support patients with low albumin or COP, respectively. In a recent study, HES 130/0.4 was able to maintain COP levels but did not significantly increase COP when administered as a CRI over 24 h to a small group of dogs with hypoalbuminemia (56). To the authors' knowledge, there are no other publications reporting the efficacy of administering colloids as a CRI rather than as a bolus. Ultimately, whether normalizing and maintaining COP should be a critical point in patient care is unclear as extravasation is at least as dependent on the integrity of the EG as it is on intravascular COP. Furthermore, synthetic colloids are not capable of carrying out all the other roles of natural colloids and specifically albumin. Therefore, they cannot be used interchangeably in the hypoalbuminemic patient.

### Summary on Physiology of Colloid Solutions

In conclusion, natural and synthetic colloids may exert greater intravascular volume expanding effect per unit administered than crystalloids in healthy patients or patients with hypovolemia. However, caution is required when administered in patients with EG damage or other causes of increased vascular permeability. Not only does increased vascular permeability reduce the expansion of volume that colloids can lead to but extravasation of the macromolecules might exacerbate edema and subsequently impair tissue oxygenation. Previous indications for use of colloids include volume expansion (including perioperative hypotension) and COP optimization. These indications should be revised considering specific conditions (e.g., intact or damaged EG), alternative therapies (e.g., vasopressors rather than fluids for drug-induced hypotension), potential harmful effects (e.g., HSA leads to anaphylactic reactions in dogs), and overall availability (e.g., canine albumin found only in limited numbers of countries).

## Hydroxyethyl Starch

### Characteristics of HES Solutions

Hydroxyethyl starch is a potato or waxy maize–derived amylopectin, modified by the substitution of hydroxyl groups with hydroxyethyl residues on the glucose subunits to increase resistance to degradation in the blood. Recent reviews described pharmacokinetics, pharmacodynamics, effects, and adverse effects of HES (2, 4, 63, 64). Preparations are characterized by the mean molecular weight (molecular size, indicated in kilodalton, kDa), molar substitution (mole of hydroxyethyl residues per mole of glucose subunit), C2:C6 ratio (locations of hydroxyethyl residues on the carbon atom of the glucose subunits) and the concentration [e.g., 6% (60 g/L) vs. 10% (100 g/L)], the carrier solution (normal saline vs. polyionic, buffered, and balanced), and the starch source [potato (e.g., HES 130/0.42) vs. maize starch (e.g., HES 130/0.4)].

Since its introduction on the market in the 1970s, HES was one of the most commonly used resuscitation fluids in people worldwide (2, 3).

### Mechanisms of Adverse Effects of HES

**Figure 1:**
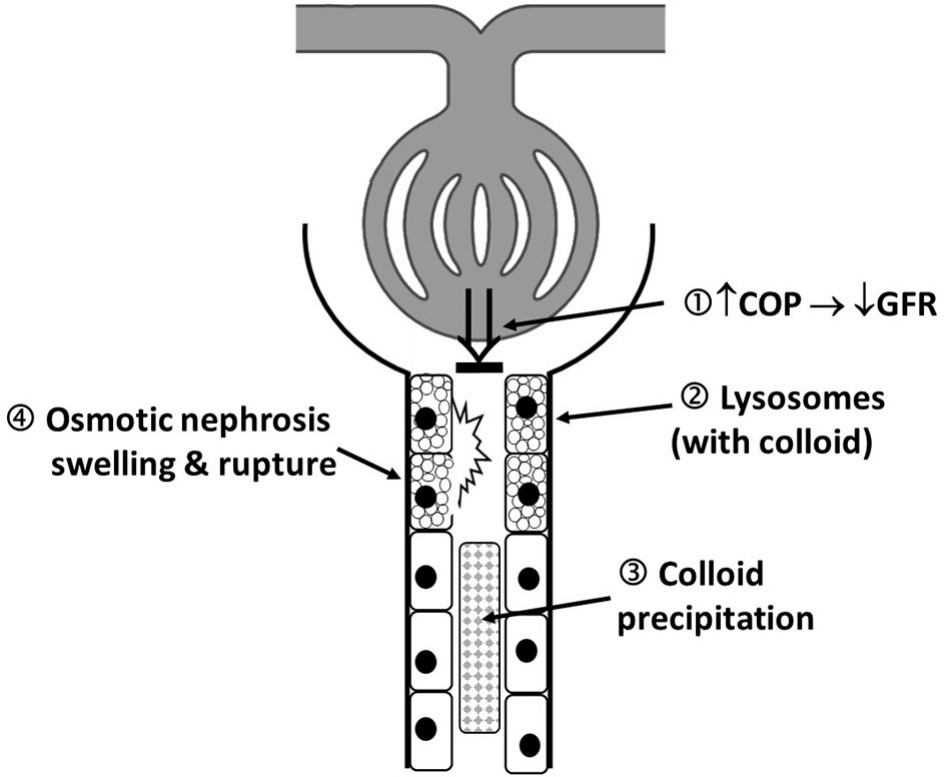
Schematic illustration of proposed mechanism of colloid-induced AKI: 1. Colloid induced increase in plasma COP that decreases filtration pressure and consequently GFR (“hyperoncotic AKI”). 2. Accumulation of colloid containing proximal tubular lysosomes, leading cellular dysfunction. 3. Local hyperviscosity and colloid precipitation, forming of occluding casts. 4. Osmotic nephrosis represents vacuolization and swelling of the renal proximal tubular cells; It can be reversible, and function restored but may also be a first step in the development of irreversible cell lesions. The above mechanisms are not mutually exclusive and may occur in combination. AKI, acute kidney injury; COP, colloid osmotic pressure; GFR, glomerular filtration rate.

HES-associated adverse effects, such as nephrotoxicity, coagulopathy, tissue storage, and therapy-resistant pruritus, were reported early after its introduction on the market (2). After intravenous administration, large HES molecules (>60 kDa) are degraded by plasma α-amylase and by the reticuloendothelial system, before they undergo glomerular filtration and urinary excretion (2). However, HES is also rapidly (within hours) taken up by a wide variety of tissues in the body, with skin and kidney being the most affected organs (65). Intracellular HES degradation is slow and incomplete, and HES deposits persist up to 10 years in the human kidney (65). A key mechanism responsible for HES-induced acute kidney injury (AKI) is osmotic nephrosis after HES uptake in renal proximal tubular epithelial cells by pinocytosis. Osmotic nephrosis is characterized histologically by a vacuolization (i.e., storage in lysosomes) and swelling of the renal tubular epithelial cells, which compromises and occludes the tubular lumen, with subsequent stasis of urine flow (66). Another mechanism is hyperviscosity-mediated injury, in which hyperoncotic tubular fluid leads to stasis of flow and obstruction of the tubular lumen (Figure 1) which has been described with 10% HES solutions (ovine endotoxemic shock model) (67). Further, renal interstitial cell proliferation, macrophage infiltration, and tubular damage are documented pathological mechanisms of HES-induced adverse effects on renal function (68, 69). In an isolated renal perfusion model, 10% HES 200/0.5 caused more tubular damage compared with 6% HES 130/0.42 and Ringer's lactate (68).

Mechanisms for HES-induced coagulopathy include dilution of clotting factors, impaired platelet function, decreased concentrations of circulating von Willebrand factor and factor VIII, impaired factor XIII–fibrin cross-linking, and enhanced fibrinolysis, with the subsequent risk of bleeding complications (2, 4, 70, 71). The pathophysiologic consequence is an impairment of different hemostatic variables (i.e., activated partial thromboplastin time) (71), which may also complicate the interpretation of coagulation abnormalities in critically ill human and animal patients (e.g., HES induced vs. disseminated intravascular coagulation-induced prolongation of clotting times).

### Adverse Effects of HES in Humans

#### Acute Kidney Injury and Mortality

The presumed beneficial effects of HES and the development of modern and putatively safer HES preparations seemed to outweigh the described adverse effects for a long time. This changed after the results of large multicenter randomized controlled trials (RCTs) in people, which compared HES and crystalloids for resuscitation of critically ill patients. Indeed, three large influential RCTs in nearly 600 patients with severe sepsis (“VISEP” in 2009), in 800 patients with severe sepsis with high illness severity (“6S” in 2012), and in 7,000 less sick intensive care patients (“CHEST” in 2012) found an increased risk of AKI and renal replacement therapy (RRT) (in all three trials), and mortality (only in “6S”) in patients receiving HES (72–74) (Supplementary Table 1). These findings challenged the risk:benefit ratio of HES. Proponents criticized these landmark trials for their “flawed” design, i.e., late use of HES in patients who were already volume resuscitated (in VISEP study patients received HES for up to 21 days) and including patients who received HES even though they were in renal failure (75). Around the same time, two smaller RCTs in 115 patients with penetrating and blunt trauma (“FIRST” in 2011) and in 156 patients with severe sepsis (“CRYSTMAS” in 2012) reported no adverse effects (76, 77). In addition, a large open-label randomized comparison between crystalloids and various colloids for the treatment of acute hypovolemia in nearly 2,900 patients with hypovolemic shock (“CRISTAL” in 2013) showed no significant difference in 28-days mortality, but a significant reduction in mortality at 90 days [34.2% (crystalloids) vs. 30.7% (colloids), *p* = 0.03] in the colloid group (70% of colloids were HES), and more vasopressor-free and ventilator-free days by day 28. The study also found no evidence that colloids increased the risk of AKI or any other serious adverse event (Supplementary Table 1) (78). Independently of these controversial RCT results, almost 90 HES-related scientific articles authored by a prominent German anesthetist and prolific defender of HES were retracted 2012 for data fabrication and lack of ethics approval (79, 80). The retraction of such a large body of work has had far-reaching effects on clinical practice and research oversight (81). Because of the RCTs demonstrating harm in critically ill patients receiving HES and the retraction of the HES-supporting scientific articles, several systematic reviews then highlighted the adverse effects of HES (82–85). As a consequence, two pharmacovigilance safety reviews of HES worldwide in 2012/2013 and in the European Union (EU) in 2017/2018 led to progressive restrictions of HES use in people (86). Since then, experts worldwide have considered HES solutions to be contraindicated in patients with sepsis or other critical illnesses, additional warnings on packaging are required in the EU, and supply has been limited to accredited hospitals after training of healthcare professionals (86). These restrictions are still a matter of debate between HES proponents and opponents. Nevertheless, between 2007 and 2014, the proportion of patients receiving colloids in human medicine decreased significantly, which was primarily due to a decrease in the use of HES, despite an overall increase in the use of human albumin (6). As most of the negative effects of HES reported in the RCTs involved patients with sepsis, in 2013 European medicines authorities requested further studies evaluating the safety and efficacy of HES in perioperative non-septic patients (86). Several smaller studies and RCTs in non-septic patients in the perioperative setting found no evidence of a nephrotoxic effect of HES (87, 88). Likewise, two recent larger, multicenter trials in 4,545 (“RaFTinG” in 2018) and 775 (“FLASH” in 2020) surgical patients also found no significant difference in mortality between patients who received HES or crystalloids and AKI in one (89), and according to the authors a reduced risk of RRT and AKI in another (90) (Supplementary Table 1). Notably, “RaFTinG” was criticized for statistical over-adjustment and “FLASH” for misrepresenting results (91, 92). In summary (and regardless of the exhaustive criticism all RCTs have been subjected to), critically ill and specifically septic patients seem to be particularly burdened by the nephrotoxicity of HES. This is exacerbated by long-term HES administration to otherwise hemodynamically stable patients. Such effects seem to be lost in less severely ill patients and specifically those presented with acute hypovolemia or those requiring perioperative fluid therapy.

#### HES Coagulopathy

In addition to the results regarding kidney failure in the aforementioned studies, HES-induced coagulopathy and hemorrhage is also a well-known adverse effect (73, 74). Despite improved physicochemical properties of newer HES products (e.g., tetrastarch), a systematic review from 2011 found HES 130/0.4 led to hypocoagulability in a dose-dependent fashion (70). This was supported in further clinical studies, where patients who received HES lost more blood and had higher need for packed red blood cell (pRBC) transfusions than patients receiving only crystalloids (74, 88). In 6S, more patients in the HES group than in the Ringer's acetate group received blood products, including packed red cells (relative risk, 1.28; 95% CI 1.12–1.47; *p* < 0.001) (74). In two recently published RCTs, HES therapy was associated with a blood loss more than twice as high as with crystalloid alone (87, 93). In contrast, a meta-analysis including 49 studies in a total of nearly 3,439 patients undergoing cardiac surgery (i.e., colloids are used as priming solution and volume replacement) could not identify evidence for a higher risk of bleeding, blood transfusion, or reoperation associated with tetrastarch compared with pentastarch, albumin, gelatin, and crystalloids. Tetrastarch was superior to human albumin in terms of blood loss and transfusion requirements (blood loss after tetrastarch vs. albumin: standardized mean difference, −0.34; 95% CI, −0.63 to −0.05; *p* = 0.02) (94).

#### Safety Recommendation in People

According to the latest recommendation from the European Medicines Agency from 2018, valid for the EU countries, HES use should be limited to initial volume resuscitation with a dose not exceeding 30 ml/kg over a period of administration not exceeding 24 h, and that kidney function should be monitored for at least 90 days thereafter. In addition, HES is contraindicated in sepsis, critically illness, burns, severe preexisting coagulopathy, and bleeding in people, among others (86).

### Adverse Effects of HES in Small Animals

HES is currently the most used and studied synthetic colloid in veterinary medicine. Guidelines or absolute contraindications on the use of HES do not yet exist for small animals. Longstanding recommendations for the use of HES in animals have been mostly extrapolated from human medicine so the recent changes in human guidelines have led to more veterinary research.

#### HES-Induced Acute Kidney Injury in Small Animals

Most of the studies of the renal effects of HES in dogs and cats have been retrospective or experimental and have yielded conflicting results (95–102) (Table 2). In one retrospective study, treatment with 10% HES 200/0.5 was a risk factor for adverse outcome, including in-hospital death or AKI, and the risk of AKI increased with increasing HES dose (97). In four retrospective studies in critically ill dogs and cats, 6% HES 130/0.4 was not associated with an increased risk for AKI (98–101), although the number of HES days was significantly associated with an increase in AKI grade within 10 days post-HES (98). Two of these studies specifically evaluated a subgroup of cats and dogs with sepsis and found no HES-associated acute kidney injury (100, 101). Interpretation of these studies is hampered by the fact that different HES types are used, the definition of AKI is not standardized, study populations are comparatively small, and the studies are retrospective in nature, indicating associations rather than causality. Further, creatinine has a poor sensitivity to detect early stages of AKI. A conclusion regarding the renal safety of tetrastarch based on these four studies is therefore limited.

**Table 2:** Clinical studies evaluating kidney injury after HES administration in dogs and cats (sorted by publication year).

**First author (year, [reference])**	**Design**	**Species**	**HES type**	**Definition of AKI**	**Measurement time points**	**Doses**	**Outcome**
Hayes et al. (97)	Retrospective, critically ill	Dogs (HES *n* = 180; CRYS *n* = 242)	10% HES 200/0.5	>2× increase in admission creatinine concentration or oliguria/ anuria of <0.5 mL/kg/h for >12 h	Blood creatinine concentrations at admission and during hospitalization.	Median bolus dose: 8.2 ml/kg/d (IQR 5.0–11.3 ml/kg/d); CRI median dose: 26 ml/kg/d (IQR 24.0–48 ml/kg/d)	HES increased risk of an adverse outcome including death or AKI (OR = 1.98, 95% CI = 1.22–3.22, *P* = 0.005)
Yozova et al. (100)	Retrospective, critically ill	Dogs (HES *n* = 86; CRYS *n* = 115)	6% HES 130/0.4	VAKI staging system	Plasma creatinine concentrations at admission (T0), and 2–13 days (T1), and 2–12 weeks (T2)	Median total dose: 86 ml/kg (range, 12–336 ml/kg); Median bolus 25 ml/kg/d (range, 12–62 ml/kg/d); median duration of administration 3.7 days (range, 1–9 d)	Compared to CRYS, HES did not result in greater increase in creatinine in critically ill dogs and in the subgroup of dogs with sepsis
Yozova et al. (101)	Retrospective, critically ill	Cats (HES *n* = 31; CRYS *n* = 62)	6% HES 130/0.4	IRIS AKI grading criteria and VAKI staging system	Plasma creatinine concentrations at admission (T0) and the maximum concentration measured at any time between 24 h after admission and discharge or death (T1max)	Median total dose: 94 ml/kg (range 26–422 ml/kg); median daily dose: 24 ml/kg/day (range 16–42) 3.7 days of administration (range 1–13d)	Compared with CRYS, HES did not result in greater increase in creatinine in critically ill cats and in the subgroup of cats with sepsis
Sigrist et al., (98)	Retrospective, critically ill	Dogs (HES *n* = 94; CRYS *n* = 90)	6% HES 130/0.4	IRIS AKI grading criteria	Serum creatinine concentration was recorded each available day until day 90	Median total dose: 69.4 ml/kg (range, 2–429 ml/kg); median daily dose 20.7 ml/kg/d (range, 2–87 ml/kg/d); median duration of administration 4 days (range, 1–16 d)	Compared with CRYS, HES did not result in greater increase in creatinine. Number of HES days was significantly associated with risk of increased AKI grade within 10 days post-HES administration
Sigrist et al. (99)	Retrospective, critically ill	Cats (HES *n* = 26; CRYS *n* = 36)	6% HES 130/0.4	VAKI staging system	% change from baseline to the last and highest creatinine within 2–10 days and from baseline to the last creatinine within 11–90 days	Mean total dose: 98.5 ml/kg (range, 8–278 ml/kg); mean daily dose: 20.1 ml/kg/d (range, 8–40.5 ml/kg/d) Median duration of administration 4 days (range, 1–11 d)	Neither administration of HES, the HES dose or number of HES days were associated with an increased risk for AKI
Boyd et al. (102)	Prospective randomized controlled blinded clinical trial in dogs prescribed a fluid bolus of at least 10 mL/kg	Dogs (HES = 21; CRYS = 19)	6% HES 130/0.4 vs. Hartmann's solution	Urine biomarkers of AKI (NGAL, cystatin C, KIM, clusterin, and osteopontin) and VAKI staging system	Concentrations of osmolality-indexed biomarkers prior to and 6, 12, and 24 h after the first study fluid bolus where compared in linear mixed-effects models. The maximum VAKI score up to 7 days during hospitalization and in-hospital mortality	Mean volume of study fluid was not significantly different between groups (HES: 23.1 mL/kg, CRYST: 25.9 mL/kg)	No differences in change over time of urine AKI biomarkers in dogs treated with 10 to 40 mL/kg HES or CRYST over 24 h VAKI scores and mortality were not significantly different between groups

*AKI, acute kidney injury; CRYS, isotonic crystalloids; HES, hydroxyethyl starch; NGAL, neutrophil gelatinase-associated lipocalin; KIM1, kidney injury molecule-1; OR, odds ratio; VAKI, Veterinary Acute Kidney Injury*.

Two experimental studies compared the effect of 6% HES 130/0.4 (bolus of ~20 ml/kg once), of other colloids, and of crystalloids on kidney injury in canine hemorrhagic shock models (95, 96). Concentrations of several plasmatic and urinary renal biomarkers were analyzed, e.g., neutrophil gelatinase-associated lipocalin, a protein expressed after renal proximal tubular damage and secreted in blood and urine. Both studies found no association between HES and biomarkers of AKI within 3 or 72 h after HES bolus, respectively (95, 96). It is unclear to what extent these results can be extrapolated to critically ill or septic dogs and cats, as acute hemorrhagic shock was induced in previous healthy dogs, and the studies were conducted over a relatively short period of time in a small group of dogs and in a defined disease model.

Prospective RCTs are needed and are currently underway. Recently, results from a randomized, blinded, clinical trial to compare different urine biomarkers (e.g., neutrophil gelatinase-associated lipocalin) of AKI in dogs receiving 6% HES 130/0.4 vs. Hartmann's solution for shock resuscitation were published (102). No differences in urinary AKI biomarkers between the HES group (21 dogs) and the crystalloid group (19 dogs) were found up to 24 h after the first fluid bolus. In addition, in the five dogs which developed AKI (i.e., three in the HES group, two in the crystalloid group), no significant difference in maximum Veterinary Acute Kidney Injury scores was found (102).

Intrarenal HES accumulation has also been evaluated histologically in dogs (95, 103). Tubular injury scores (based on damaged epithelial cells and presence of sloughed cells within tubular lumen) and the degree of renal tubular microvesiculation (vesicles within renal epithelial cells) were not significantly different between dogs receiving 6% HES 130/0.4 compared with dogs receiving whole blood or isotonic crystalloids in an experimental hemorrhagic shock model (95). In contrast, retrospective histopathological examination of kidney tissue from critically ill dogs revealed that the cumulative dose of 6% HES 670/0.75 was positively associated with the severity of renal tubular vacuolization (103). It is worth mentioning that osmotic nephrosis is not specific to HES administration but can be due to other drugs (e.g., mannitol, glucose, intravenous immunoglobulins), primary renal diseases, and postmortem autolysis (66). Immunohistochemical evaluation with specific HES antibodies would be ideal to provide a reliable diagnosis of HES-induced osmotic nephrosis.

#### HES-Induced Coagulopathy in Small Animals

Several *in vitro* and *in vivo* studies in healthy dogs and cats (104–108) have shown a dose-dependent and transient coagulation impairment after *in vitro* dilution of blood or IV administration of HES. Hemostasis assessment was performed predominately by whole blood platelet function analyses and viscoelastometric coagulation analyses (e.g., rotational thromboelastometry). Platelet function, speed of clot formation, and clot firmness were more impaired after HES than after crystalloids using similar dilutions (104–108). However, when clinical doses of HES and isotonic crystalloid (i.e., 10 ml/kg HES vs. the 3- to 4-fold volume of crystalloid) were compared *in vitro* (blood from healthy dogs) (107) or *in vivo* (experimentally induced canine hemorrhagic shock model), no difference was found (109, 110). Different results were obtained in two other studies which used thromboelastometry. In one hemorrhagic shock model, the administration of 20 ml/kg of 6% HES 130/0.4 was associated with hypocoagulability beyond effects of hemodilution (i.e., 80 ml/kg crystalloids) (111). Likewise, in critically ill dogs with spontaneous hemoperitoneum, more impaired coagulation was found after 10 ml/kg of 6% HES 130/0.4 compared with a 3-fold volume of crystalloid (112). However, in this study, parameters for clot firmness remained within reference ranges and no statistically significant differences in standard coagulation tests were found between the HES and crystalloid group (112). The clinical relevance of the aforementioned changes has not yet been conclusively determined because actual bleeding tendencies have been evaluated in only one study in dogs. In this study, no clinical bleeding was detected after 10 ml/kg of 6% HES 600/0.75 administered to healthy anesthetized dogs. HES coagulopathy appears to last <3 h [15 ml/kg of 6% tetrastarch (106)] and <24 h [20 ml/kg of 6% hetastarch (113)], respectively, after a single bolus in healthy dogs.

Additional findings in several *in vivo* studies were a significant decrease in hematocrit and platelet count after a HES bolus (106, 112) which was disproportionate to the administered volume of HES (i.e., greater decrease in hematocrit after HES than after crystalloid). The reason for this disproportionate decrease in hematocrit has been proposed to be mobilization of plasma volume previously retained within the endothelial glycocalyx (25).

### Recommendations for HES Use in Small Animals

In general, HES should be avoided if the animal responds already to crystalloid or other therapy. HES is not a replacement for albumin and in patients with severe hypoalbuminemia, natural colloids (such as plasma products or albumin) should be considered. In complex cases (such as sepsis or septic shock), therapy is multimodal, and potentially natural colloids and/or vasopressors are necessary. The current evidence favors a very limited risk for HES-associated AKI in dogs and cats when administered in small doses (<20 ml/kg) for short periods of time. Previously healthy animals with acute hypovolemia (e.g., trauma-induced hemorrhage) could inconsequently tolerate administration of HES in the initial resuscitation phase, and HES can be considered if substantial amounts of crystalloids are required. The necessity for colloids is questionable as isotonic or hypertonic crystalloids usually provide adequate resuscitation endpoints in such patients. Administering HES to hemodynamically stable patients and patients with pre-existing azotemia is not recommended.

Based on extrapolated data from people, resuscitating critically ill and specifically septic patients with HES might be detrimental especially in high doses and for long periods of time. Furthermore, as mentioned previously, it might also be futile because damage to their EG exacerbates extravasation of any fluid. Currently, there is no evidence that HES has any disadvantages in veterinary patients with sepsis; however, there are no proven benefits to date either.

The effects of HES on different coagulation tests seem to be mild in dogs without preexisting coagulopathy, and mild to moderate in those with preexisting coagulopathy when using doses <20 ml/kg. Given the evidence in human medicine (definite increased risk of bleeding) and the unclear association between standard laboratory tests and actual clinical bleeding risk in dogs, monitoring of coagulation parameters and clinical bleeding as well as hematocrit and platelet count should be considered if the HES dose is ≥10 ml/kg in patients with potential preexisting coagulopathy, anemia, or thrombocytopenia (112).

In contrast to human medicine and specifically high-income countries, veterinarians need to incorporate financial considerations in their decision-making. Furthermore, prompt expensive therapy might be hindered by owner indecisiveness (whether on financial or personal grounds) or availability. In such instances, HES could be considered as a short-term solution in patients where more costly therapies (i.e., allogenic albumin and plasma products) are unaffordable or while decisions are made. Hydroxyethyl starch should be used as a bolus at the lowest possible dose. Under consideration of the existing literature, the authors recommend a dose for 6% tetrastarch of 5–10 ml/kg for dogs and 3–5 ml/kg in cats over 10–15 min in states of hypovolemia. This can be repeated if needed up to a total dose of 20 ml/kg. The authors do recommend against the use of HES as a CRI, as the effects on COP is negligible (56) and a significant association was found between days of administration of HES and AKI grade (98). Kidney function should be monitored after HES exposure—according to human studies up to 90 days after exposure. Veterinary clinicians should consider discussing the potential adverse effects of HES with owners of septic patients.

## Gelatin

### Characteristics of Gelatin Solutions

Gelatin solutions contain bovine gelatin hydrolysate, chemically modified to increase solubility. Gelatin preparations have a mean molecular weight of ~30 kDa. As this is below the renal threshold for glomerular filtration, most of the gelatin is excreted into the urine within minutes of infusion. Thus, the volume effect (70–80%) and the duration of volume expansion (2–3 h) are expected to be more limited compared with HES (114). Gelatin is available as a 4% succinylated gelatin solution in normal saline or in a buffered, polyionic isotonic carrier solution (Table 1). Gelatin was withdrawn from the US market in 1978 due to concerns of increased blood viscosity and coagulation impairment (115). In addition, animal experiments suggest harmful effects on kidney structure and function similar to the effects seen with HES (116, 117) (Figure 1). In countries where gelatin is still licensed (e.g., Germany, Switzerland), it is contraindicated in severe renal failure. However, there is no clear dose or maximum daily limit, and the recommendation on the package insert states that the “maximum daily dose is determined by the degree of hemodilution.” Due to the restrictions in HES use, gelatin could potentially become an alternative synthetic colloid in many European countries.

### Adverse Effects of Gelatin in People

Significantly fewer clinical studies on gelatin exist in human medicine as compared with HES. Despite the lack of RCTs evaluating the risks and benefits of intravenous gelatin in people, clinicians in human medicine seem to have increased their use of gelatin following the HES restrictions from 2013 and 2018 (118, 119). There is nonetheless evidence that gelatin might be at least as detrimental to kidney function as HES with a more limited understanding of the underlying mechanism. In a prospective study in people with severe sepsis, AKI occurred in 70% of patients receiving HES and in 68% of patients receiving gelatin, vs. 47% of patients receiving crystalloids (120). In a systematic review and meta-analysis of gelatin-containing plasma expanders vs. crystalloids and albumin in people, the authors concluded that gelatin solutions increase the risk of anaphylaxis, and may increase bleeding, renal failure, and mortality. The authors recommended against the use of gelatins (119). A prospective currently ongoing study, Gelatin in ICU and Sepsis (GENIUS), is a double-blind RCT investigating the efficacy and safety of gelatin as opposed to crystalloid administration in two parallel groups of patients with severe sepsis/septic shock (NCT02715466). Results from that study are expected end of 2021.

### Adverse Effects of Gelatin in Small Animals

Gelatin seems to be rarely used in small animals. According to an international survey about the use of colloids in small animals from 2016, gelatin was used by only 4% of the respondents (in contrast to 85% who used HES), although it was the main synthetic colloid used by 24% of respondents from the UK (5). This finding might be the result of reduced availability of HES products in specific countries (e.g., the UK recalled all HES products from the market in 2013 after the EMA review was initiated). Therefore, veterinarians were forced to use alternative licensed options such as gelatins. Only a few reports of the effects on coagulation and renal injury in dogs, and no reports in cats have been published (95, 111, 121–123).

In regard to its effects on coagulation, experimental studies have shown that 20 ml/kg of 4% gelatin leads to impaired platelet function (111), but that 10 to 20 ml/kg of 4% gelatin had negligible effects on thromboelastography and plasma coagulation (111, 123).

Regarding its renal effects, gelatin led to a marked increase in urine and plasma renal biomarkers, e.g., a 33-fold increase in urinary neutrophil gelatinase-associated lipocalin and a 5.9-fold increase in the urinary concentration of clusterin (a renal glycoprotein induced in response to a variety of injuries including ischemia/reperfusion) (95). Notably, this increase was significantly more than after the same dose of 6% tetrastarch. The same study found prominent renal epithelial cell microvesiculation in all dogs receiving 4% gelatin, although these were not sufficiently severe to meet the definition of “osmotic nephrosis” (95). As mentioned earlier, these data cannot be directly extrapolated to critically ill dogs and cats. However, preexisting renal damage in critically ill and septic patients favors the pathogenesis of osmotic nephrosis-related AKI, and certainly predisposes patients even more to gelatin-induced AKI.

### Recommendations for Gelatin Use in Small Animals

According to the results of a small number of studies, the administration of gelatin significantly impairs platelet function and is associated with a high risk of renal injury in dogs. Because the volume-expanding effects of gelatin are inferior to HES, yet the risk of renal injury is significant, it is not appropriate to use gelatin as an alternative to HES.

## Dextrans

### Characteristics of Dextran Solutions

Dextrans are neutral, high-molecular-weight glucose polysaccharides synthesized from sucrose by the bacteria *Leuconostoc mesenteroides* or *dextranicum* (114). Commercially available dextran solutions have an average molecular weight of either 70 kDa (dextran-70) or 40 kDa (dextran-40). Concentrations used are 3, 6, and 10%, and they are mixed in either isotonic or hypertonic electrolyte solutions (Table 1) (1, 124). Dextran is either excreted by the kidneys or metabolized by hepatic dextranase and the glucose monomers are ultimately oxidized to carbon dioxide and water (1). Dextran-40, consisting of smaller molecules, has more particles per unit weight than dextran-70, and hence it is more osmotically active. However, its smaller molecular fractions pass faster through semipermeable membranes of the capillaries into the interstitial space and through glomeruli into the urine. Thus, the volume expansion effect of 10% dextran-40 is 150% with a duration of action of 3–5 h, while the volume effect of 6% dextran-70 is 100% with a duration of action of 6–8 h (up to 20 h), due to its larger molecular fractions and prolonged excretion (125). Hypertonic 10% dextran-40 leads to an initial volume expansion of up to 200% (114).

Major adverse effects in people include anaphylactoid reactions, AKI, and impaired coagulation. Several theories about the mechanism for dextran-induced AKI have been proposed, with some of these mechanisms shared with other colloids. These include an increase in plasma COP with subsequent decrease in glomerular filtration pressure and reduced GFR; precipitation of dextran within the renal tubules forming occluding casts and osmotic nephrosis (66, 126, 127) (Figure 1). Dextran is also known to impair coagulation, with a subsequent increased risk of bleeding (128). The underlying mechanisms are not well-understood, but bare similarities with other synthetic colloids. These include inhibition of platelet aggregation by reducing the activity of factor VIII, von Willebrand factor, and the glycoprotein IIb/IIIa receptor, affecting fibrinogen polymerization by co-binding, and enhancing fibrinolysis by increasing the circulating levels of tissue type plasminogen activator, and decreasing the levels of plasminogen activator inhibitor-1 (128–130).

According to a 2010 international study (3), the use of dextran in people was widespread in Scandinavia. However, it remained the least used colloid, accounting for only 3% of the total international responders. Dextran has been withdrawn from the human market in several countries (i.e., Germany, Switzerland) but seems to replace HES in others (e.g., Sweden) (131).

### Adverse Effects of Dextrans in People

Anaphylactoid reactions after dextran are the result of preformed endogenous anti-polysaccharide antibodies, which cross-react with dextran molecules, causing an immune reaction (114, 132). These severe reactions are prevented by “hapten inhibition” using dextran 1, which binds dextran-reactive antibodies and forms inactive complexes. The dextran 1, which has been used for hapten inhibition since the 1980s, can be administered a few minutes before the infusion of the main solution of dextran. A 35-fold reduction in anaphylactoid reactions after introduction of the prophylactic use of hapten inhibition was reported in a study from Sweden from a decade after its introduction (133).

Numerous cases of AKI after administration of 10% dextran-40 have been published (69, 134–137). While these older studies have shown an increased risk of AKI with the use of dextran-40, studies of dextran-70 are few, probably because of its limited use worldwide. However, existing data suggest that dextran-70 is also a potential risk factor for the development of AKI in patients with septic shock (138, 139).

Impairment of *in vitro* coagulation and increased bleeding after dextran-70 has also been found in different patient groups (131, 138, 139). Due to this particular side effect, dextran was used as a prophylactic agent against postoperative thrombo-embolism in the 1980 (140).

### Adverse Effects of Dextrans in Small Animals

In the 1990s, hypertonic saline with 6% dextran-70 (HSD) preparations were studied in different canine shock scenarios, such as septic, endotoxic, hemorrhagic, and traumatic shock, and gastric dilatation volvulus. As part of developing “small volume resuscitation” strategies, the volume effects of dextran were compared with isotonic crystalloids (141–146). In all mentioned studies, bolus therapy with 4–5 ml/kg of HSD solution was found to be a more effective resuscitation solution compared with an isotonic crystalloid, and 8–10 times less volume was necessary to reach similar cardiovascular endpoints. To the authors' knowledge, there are no reports about dextran-induced anaphylactoid reactions or AKI in dogs. Two studies evaluated hemostasis after dextran-70 in dogs and found a dose-dependent impairment in different hemostatic variables (such as plasma coagulation assays, platelet numbers, factor VIII coagulant activity, von Willebrand factor antigen concentration, and platelet function and buccal mucosal bleeding time) (122, 147). It is likely that as the preference of synthetic colloids shifted toward HES solutions, clinical studies of dextrans in dogs or cats were no longer performed. This may change in the near future. In a recent experimental study in dogs, resuscitation after hemorrhagic shock with HSD showed a similar hemodynamic response compared with Ringer's lactate at 10 times the volume of HSD, but HSD showed superior efficacy in organ protection (kidneys, lungs, and liver) (148).

### Recommendations for Dextran Use in Small Animals

Dextran-70 seems to be efficient for volume resuscitation in dogs, especially when combined with hypertonic saline. Other than coagulation impairing effects, adverse effects are largely unknown, and as for other colloids, nephrotoxic effects are likely. There are insufficient data in dogs, and no data in cats, to make a recommendation for dextran use.

## Albumin

### Characteristics of Albumin Solutions

Solutions of human serum albumin are prepared from pooled human serum, with sterile water as the carrier. The solutions available for intravenous use contain no preservative, and pathogen inactivation is performed by pasteurization at >60°C for 10 h (149, 150). It is usually available as 5, 20, and 25% solutions, with availability varying geographically. Albumin accounts for ~50% of the plasma protein content and is responsible for about 80% of the plasma COP (151, 152). A near exponential relationship exists between albumin concentrations and *in vitro* COP, which is explained by the Gibbs–Donnan effect (153). The negative charges on amino acids within albumin attract cations (which have and osmotic effect), leading to a disproportional increase in COP (153). Thus, a 5, 12.5, and 25% HSA solution exerts a COP of ~20, ~95, and >200 mmHg, respectively (153).

Albumin is incorporated into the EG, where it contributes to vascular integrity, and normal capillary permeability. In addition, albumin has antithrombotic, antioxidant, and anti-inflammatory properties (55, 154). Hypoalbuminemia occurs as a result of many critical diseases such as systemic inflammation, sepsis, burns, liver failure, protein-losing enteropathy/ nephropathy, and severe prolonged malnutrition. Severe hypoalbuminemia can lead to gastrointestinal complications (i.e., gastric and intestinal edema, gastrointestinal ileus), effects on coagulation (i.e., hypercoagulability, increased platelet aggregation), delayed wound healing, and consequences secondary to the decreased COP (i.e., tissue edema, body cavity effusions) (152). Furthermore, hypoalbuminemia has been associated with high morbidity and mortality rates in human and veterinary patients (100, 155, 156). A meta-analysis in critically ill hypoalbuminemic human patients found that each 10 g/L decrease in serum albumin concentration increased the odds of mortality by 1.37 (156). Nevertheless, it remains unclear whether these effects are a direct result of the albumin deficit, in which case albumin replacement would be beneficial, or if it is merely a marker for the disease severity, and albumin optimization would not lead to improved patient outcomes.

### Use of Albumin in People

Albumin is commonly used for fluid resuscitation in people (157–159). Usually, 5% HSA (and more rarely 20% HSA) is administered as a second-line treatment after crystalloids (24, 159, 160). Another indication is correction of significant hypoalbuminemia. Albumin is not only used in conditions requiring COP support. In severe liver diseases such as cirrhosis albumin is recommended for its non-oncotic properties such as antioxidant, scavenging, immune-modulating, and endothelial protective functions (161). Albumin solutions are used in various settings and conditions including priming cardiopulmonary bypass circuits, therapeutic plasma exchange, nephrotic syndrome, and pancreatitis (159). They are not indicated as a nutritional source to raise serum albumin *per se* without hypovolemia (159). Anaphylactoid reactions have been reported in <0.1% of recipients (162).

The use of albumin in critical care has generated controversy for decades. In 1998, a Cochrane systematic review of 30 small RCTs suggested harmful effects (i.e., increased mortality) of albumin as compared with crystalloids for volume replacement in critically ill patients (163). The impact of this meta-analysis was dramatic and led to a substantial reduction in the use of albumin in some countries. In 2004, the “SAFE” study was published, a RCT which compared mortality in 6,997 critically ill patients with hypovolemia who had received 4% albumin *or* saline for intravascular-fluid resuscitation (164). A small volume sparing effect was observed during the first 4 days in the albumin group (ratio of albumin to saline was ~1:1.4), but no significant difference was found in 28-days mortality, length of hospitalization, or organ dysfunction. Consequently, albumin was considered to be “safe,” but that it did not offer any advantage over saline. Subgroup analyses revealed that patients with severe sepsis treated with albumin tended to show a better survival, although the difference did not reach statistical significance (*p* = 0.09) (164). In 2014, the “ALBIOS” trial was conducted in 1,818 patients (158). This RCT aimed to answer the question of whether in patients with severe sepsis or septic shock, administration of 20% HSA to maintain a serum albumin level ≥3 g/dl (≥30 g/L) reduces all-cause mortality at 28 days compared with no albumin. Similarly to the SAFE study (164), there was no significant difference in either the 28- or 90-days mortality or total organ failure scores. Patients who received albumin had a lower daily positive fluid balance over the first 7 days (158).

Despite the theoretical benefits of albumin and efforts to translate this into positive patient outcomes (158, 164), no RCT has yet demonstrated a significant advantage of albumin over other types of fluid, including crystalloids (165). Nevertheless, an increase in albumin use in adult intensive care patients was found recently (6). Albumin appears to have replaced HES as a resuscitation solution (6, 166). This is potentially associated with the Surviving Sepsis Campaign from 2016 which recommend volume replacement with albumin (instead of a synthetic colloid) when patients require “substantial” amounts of crystalloid during hemodynamic stabilization (167).

### Adverse Effects of Albumin in Small Animals

In small animals, intravenous albumin is mostly used to correct hypoalbuminemia and COP. Sources of albumin in dogs and cats include CSA, allogenic plasma products, and HSA. No feline-specific albumin products are available (Table 1). Canine albumin (e.g., lyophilized canine albumin) manufactured in the USA is currently not ubiquitously available. Consequently, some veterinarians are forced to use HSA products if they wish to administer concentrated albumin to dogs and cats. Human albumin shares ~80% structural homology with canine albumin (168); therefore, allergic reactions are possible. Anaphylactic and other immune reactions, and life-threatening complications have been reported in dogs after administration of 5, 10, and 25% HSA (169–173). Relevant study results are summarized in Table 3 and are explained in more detail later.

**Table 3:** List of small animal publications using human serum albumin solutions and its doses.

**Author (year [references]**	**Species**	**Setting**	**Albumin concentration**	**Total dose**	Dose per hour^*^	**Duration of administration**	**Adverse events**
Mathews and Barry, (174)	64 dogs 2 cats	Retrospective study, critically ill	25%	Mean: 1.25 g/kg	0.025–0.43 g/kg/h	Range 4–72 h	Facial edema in 2 dogs
Trow et al. (173)	73 dogs	Retrospective case series, critically ill	10%	Median 1.4 g/kg (range 0.1–6)	~0.12 g/kg/h	12 h	23% acute adverse reactions (mild: tachypnea, tachycardia, increased temperature, peripheral edema, and ventricular arrhythmias; severe: coagulopathies, cardiac arrest). 4% delayed complications
Vigano et al. (175)	418 dogs 170 cats	Retrospective study, critically ill	5%	Mean: 1 g/kg/day	0.1 g/kg/h	Dogs: Median 96 h (range 28–264 h) Cats: Median 72 h (range 48–168 h)	No acute severe adverse reaction. Minor acute adverse reactions (diarrhea, hyperthermia, and/or tremors) in 43.5% dogs and 36.5% cats (no specific treatment)
Powell et al. (172)	2 dogs	Case series, critically ill	5%	Dog 1: 1.4 g/kg Dog 2: 1.3 g/kg	0.35 g/kg/h 0.43 g/kg/h	4 h 3 h	Type III hypersensitivity reaction (leukocytoclastic vasculitis and dermal antigen-antibody complexes) 8-16 days after exposure
Horowitz et al. (176)	22 dogs + 17 negative control dogs	Retrospective study, septic peritonitis	25%	Mean: 2.55 g/kg (range: 0.95–6.38)	n/a	Mean: 39.2 h (range: 11–98 h)	No evaluation for adverse reactions
Loyd et al. (177)	21 dogs	Retrospective study, PLE	25%	Dose: 0.5 g/kg	~0.16–0.25 g/kg/h	2–3 h	2/21 acute reaction; 1 dog euthanized 2/21 delayed reaction; 1 euthanized
Vigano et al. (178)	40 cats + 20 control cats	Prospective study, critically ill	5%	Mean: 0.72 g/kg (range: 0.5–1)	0.07–0.1 g/kg/h	Mean: 7 h (range: 5–10 h)	No acute or delayed adverse reaction
Mazzaferro et al. (171)	2 dogs	Case series, septic peritonitis	25%	Dog 1: 1.5 g/kg Dog 2: ~2.6 g/kg	0.13 g/kg/h 0.33 g/kg/h	12 h 8 h	Delayed type III hypersensitivity reaction with AKI; euthanasia
Martin et al. (179)	14 critically ill + 2 healthy dogs 21 critically ill + 47 healthy dogs	Prospective, healthy and critically ill	25%	Healthy: 1st time 0.5 g/kg 2nd time 0.25 g/kg Critically ill: Median: 1.3 g/kg (0.45–11.8)	0.25 g/kg/h 0.125 g/kg/h 0.2 g/kg/h	2 h 1 h	Critically ill: transient fever in 1 dog, no other acute or delayed adverse reaction Healthy: 1st time: facial edema in 1 dog day 8 2nd time: acute adverse reaction in both dogs
Cohn et al. (169)	9 dogs	Prospective study, healthy	25%	1st time: 2.5 g/kg (9 dogs) after 5 weeks 2nd time: 2.5 g/kg (2 dogs)	0.66 g/kg/h	Mean: 3.75 h (range 3–4.5 h)	1/9, acute hypersensitivity (1st time) 2/2 acute hypersensitivity (2nd time) 2/9 urticaria/ edema after 2 weeks (1st time) 9/9 developed anti-HSA antibodies
Francis et al. (170)	6 dogs	Prospective study, healthy	25%	0.5 g/kg	0.5 g/kg/h	1 h	6/6 Delayed type III hypersensitivity reaction

* *Doses were converted into grams according to data in the publications; PLE, protein loosing enteropathy; h, hours*.

In 2007, two research groups observed profound adverse reactions after 25% HSA was administered to healthy, non-hypoalbuminemic dogs (169, 170). Cohn et al. (169) administered 25% HSA twice, 5 weeks apart, to nine healthy dogs. Anaphylactic shock (collapse, hypotension, hypothermia, tachypnea, vomiting, and diarrhea) occurred within minutes in one out of nine dogs after the first dose of HSA, and in two out of two dogs receiving a second dose, while the second HSA infusion for the remaining seven dogs was abandoned. Furthermore, two out of nine dogs developed urticaria and edema 1 week after administration, and all developed anti-HSA antibodies. Similarly, Francis et al. (170) administered 25% HSA to six healthy dogs. One out of the six developed anaphylactic shock, and all dogs developed a delayed type III hypersensitivity including lameness, edema, vasculitis, and vomiting. Two of the dogs died at 21 and 28 days after HSA administration, despite intensive care treatment. These two dogs developed disseminated intravascular coagulation, AKI, and pulmonary edema. All six dogs were found to have anti-HSA antibodies (170).

Eight publications have reported the effects (including adverse effects) of administration of HSA in critically ill hypoalbuminemic dogs and cats in a clinical setting (171–175, 177–179). Human serum albumin resulted in increased serum albumin concentrations and COP (173, 174, 176, 178) and increased systemic blood pressure (174). Two studies reported higher serum albumin concentrations in survivors after HSA administration (173, 176) and in one study the magnitude of serum albumin increase was positively associated with survival (173). All but one of the aforementioned studies (171–175, 177, 179) reported adverse reactions in some patients, of which a few were severe or fatal. One prospective study in 40 cats reported no adverse effects after administration of 5% HSA (178). Most studies were retrospective in nature and adverse effects of HSA may have remained undetected or unrecorded. Furthermore, the definition and recognition of adverse reactions is not uniform between studies.

The available literature suggests that administration of HSA induces severe adverse reactions predominantly in healthy, normoalbuminemic dogs and may induce only mild or no adverse reactions in critically ill patients. Several possible explanations for this discordant response have been proposed. Supraphysiologic concentrations of albumin might be partly responsible for acute adverse reactions in healthy normoalbuminemic dogs. Therefore, administration of HSA over a longer period (e.g., 12–24 h) or administration of diluted albumin solutions may result in fewer immunologically mediated adverse effects (170, 175, 178). Further, it is possible that deterioration of the clinical condition or death in critically ill patients have been incorrectly attributed to the underlying disease, rather than to an adverse reaction (170). It is also possible that some hypoalbuminemic dogs given HSA may not have survived long enough to develop a type III hypersensitivity reaction (170). Lastly, it is speculated that critically ill dogs may be immunosuppressed (due to the severe underlying disease) and therefore not respond as strongly to HSA as healthy immunocompetent dogs (169, 170). This was challenged by a recent case series of two critically ill hypoalbuminemic dogs with septic peritonitis receiving 25% HSA (171). These dogs developed a delayed type III hypersensitivity reaction with AKI, hypoalbuminemia, proteinuria as well as immune complex deposition and vasculitis. As the use of HSA increases, more reports of allergic reactions might be expected to emerge thus proving a fuller picture on HSA safety in animals.

Both healthy (169, 170, 179) and critically ill dogs (179) developed anti-human albumin antibodies within 7 days after administration of 25% HSA. Antibody concentrations were highest 4–6 weeks after HSA administration in the critically ill dogs. Interestingly, one study reported that 7% of dogs had anti-HSA antibodies without previous HSA infusion (179). The latter could be attributed to cross-reaction with anti-bovine albumin antibodies that developed when dogs were exposed to bovine albumin through vaccination or ingestion (180). This not only shows that HSA albumin should not be administered over longer periods of time (several days), but also some patients might be previously sensitized to the molecule and show reactions on initial administration.

Canine serum albumin was evaluated in two prospective, small-scale, clinical studies. One study evaluated 14 dogs with septic peritonitis and found increased serum albumin concentrations, COP, and Doppler blood pressure 2 h after CSA administration. No acute or delayed adverse events were found (181). In a second study (presented in an abstract form to date), six healthy Beagles received CSA at a dose of 1 g/kg at three different time points (on days 1, 2, and 14). Dogs were monitored for evidence of transfusion-associated complications during the infusions and at 1, 2, 12, and 24 h after each infusion. The follow-up period was 28 days. The authors concluded that repeated infusions appeared safe, with no adverse changes to physical examination, hematologic, or biochemical parameters (182).

### Recommendations for Albumin Use in Small Animals

In contrast to people, albumin solutions in small animals have been mainly used to increase and maintain plasma albumin concentrations (CRI over hours and days) in patients that may or may not be cardiovascularly unstable. Only one study to date has shown an association of albumin administration and increased patient survival (173). Immediate and delayed adverse reactions in some critically ill dogs have been observed in several studies, with some of them proving fatal (169–172, 174, 177, 179). Critical illness does not seem to be protective against adverse immune reactions to HSA solutions. Cats seem to be less sensitive to HSA transfusions compared with dogs; however, this is based on only three studies with a total of 211 cats (174, 175, 178). Due to the potential adverse effects, HSA should be considered only for severely hypoalbuminemic patients in which the use of isotonic crystalloids alone carries a high risk of edema and/or in which hypoalbuminemia itself is a greater risk factor than the potential adverse effects of HSA. Autologous albumin products should be used when possible. Intravenous HSA or CSA is not a substitute for a proper nutritional management in critically ill patients.

Depending on the hydration status of the patient, albumin administration may be considered at plasma albumin concentrations below 10–15 g/L (1.0–1.5 g/dl) (173–176, 178). Albumin supplementation should discontinue once the serum concentration has reached 20–25 g/L (2.0–2.5 g/dl) (152). The recommended formula for calculation of the albumin dose is dose albumin (g) = (desired albumin g/L – patient albumin g/L) × 0.3 × kg body weight (171) over 12–24 h. In the authors' experience, this formula overestimates albumin requirements, so we recommend re-evaluating plasma albumin concentration after 50% of the calculated dose and adjusting requirements accordingly. Alternatively, a dose of 2 g/kg over 10 h is recommended (183). Some authors prefer to administer diluted HSA (5 or 10%) which may be better tolerated due to a more physiological COP (173, 175, 178), but there is no conclusive proof for fewer adverse effects compared with the more concentrated form. The described length of administration varies between authors from 4–6 h (184) to 12 h (173) to several days (174, 175). Based on current literature, it is not clear whether the length of administration, the total dose, or both have an influence on delayed adverse reactions. Supraphysiological concentrations are not desired as it might lead to suppression of hepatic albumin synthesis (152).

## Allogenic Plasma Products

### Use of Allogenic Plasma Products in People

The two major indications for administration of fresh frozen plasma (FFP) in people are to prevent hemorrhage and to stop active bleeding (185). Common clinical scenarios involving the use of FFP include liver failure, reversal of anticoagulant toxicity, temporary treatment of coagulation factor deficiencies, cardiopulmonary bypass surgery, disseminated intravascular coagulation, and thrombotic thrombocytopenic purpura (185). Its use has also been reported in patients with acute pancreatitis, uremic syndrome, and severe burns (185). Furthermore, FFP is a component of “massive transfusion” protocols and used in patients with traumatic coagulopathy. Substantial controversy surrounds the indications and efficacy of administration of FFP in people (186–195). While the evidence of its efficacy in preventing or stopping bleeding is somewhat robust, other less common indications are not evidence based.

### Use of Allogenic Plasma Products in Small Animals

In veterinary medicine, FFP is most commonly used to prevent or stop bleeding (196). However, its indications extend beyond treating or preventing coagulopathies. Fresh frozen plasma is administered as a means of albumin supplementation as well as a volume expander (197, 198). It is specifically advocated to prevent further dilution of albumin and clotting factors that can result from the administration of large volumes of crystalloids or synthetic colloids. Clear guidelines for the use of FFP in veterinary patients are lacking (196). To the authors' knowledge, there are no studies investigating the effects of FFP specifically when used for fluid resuscitation.

Several retrospective studies have documented the reasons for FFP administration in small animals. In a 1999 study, 74 dogs received FFP to provide albumin in 63%, coagulation factors in 47%, α-macroglobulin in 10%, and immunoglobulins in 13% of patients (197). Another retrospective study compared the use of FFP in 283 dogs and 25 cats between two decades (1996–1998 and 2006–2008) (198). The main reasons for FFP use were coagulopathies, hypoalbuminemia, and acute pancreatitis (i.e., provision of α-macroglobulin). In the later decade, significantly more FFP was used for the treatment of coagulopathies, while significantly less FFP was used to counteract hypoalbuminemia or support patients with acute pancreatitis. Fresh frozen plasma has been advocated for administration to people with pancreatitis, and, by extrapolation, to dogs (199). The rationale for the use is to supplement plasma α-macroglobulin. This is a broad-acting protease inhibitor that prevents intravascular damage caused by circulating activated pancreatic proteases. Alpha-macroglobulin becomes depleted during the systemic inflammation (200). A multicenter RCT in people from 1991 demonstrated no benefit of administering high-volume FFP to patients with acute severe pancreatitis (201). Hence, the practice to use FFP in people with pancreatitis has long been overturned. One retrospective study compared the outcome of dogs with pancreatitis that had been or had not been given FFP (202). In that study, dogs that had received FFP had a higher mortality, and no benefit was apparent. As the authors did not control for the severity of illness, and FFP is more likely to be administered to more severely affected patients, this might have biased the results (202). Two recent retrospective studies evaluated FFP transfusions in cats. One study in 36 cats found that coagulopathies were the most common reasons for FFP transfusion (94% of cats) (203). The other study evaluated FFP transfusions in 121 cats and found coagulopathies (83%), hemorrhage (35%), and hypotension (25%) to be the main reasons for administration (204). Median doses reported in dogs are 14 ml/kg (small dogs) and 5 ml/kg (large dogs), due to the administration per unit or ½ unit per dog (197). Another study reported median doses of 15–18 ml/kg (198). One study reported median doses of 6 ml/kg in cats (204).

Two retrospective studies evaluated effects of FFP transfusion in dogs. Median prothrombin time and activated partial thromboplastin time were significantly decreased after plasma transfusion. Notably, pre- and post-transfusion serum albumin and COP were not different in patients receiving FFP. In addition, no association was found between the volume of infused FFP and outcome (198). Another study focusing on adverse effects of blood product administration in dogs reported 8% mild adverse reactions after FFP transfusions (205) compared with 22% after pRBC administrations. This contrasts with the findings in people where transfusion reactions after administration of FFP are more common than after pRBC transfusions and include life-threatening acute lung injury, and circulatory overload (185). The aforementioned retrospective studies also evaluated effects of FFP administration in cats. In one study on 36 cats, coagulation parameters seemed to improve in 90% of the cats after transfusion (203). Fifteen percent of the cats experienced potential transfusion reactions, including respiratory signs, fever, and gastro-intestinal signs. In the other study (121 cats), Doppler blood pressure increased, and they were significantly less likely to be coagulopathic after transfusion. Similarly, 16% of the cats had possible reactions, such as increased body temperature (64% of cats with reactions) and tachypnea/dyspnea (47% of cats with reactions) (204).

Recently, cryopoor plasma (CPP), a byproduct of the production of cryoprecipitate, has been used as an albumin source (206–208). The albumin concentration in canine CPP is significantly higher than in FFP, suggesting that it may be a possible treatment for hypoalbuminemia in dogs while avoiding volume overload (207). A retrospective study of 10 critically ill dogs found that plasma albumin increased after a CPP CRI (206). The dogs received a median total dose of 31 ml/kg CPP over a median duration of 16 h and a mean rate of administration of 1.8 ml/kg/h. In a recent case report (a 44-kg dog with septic shock secondary to enterectomy dehiscence), CPP was used as a source of canine albumin and intravascular volume. In this case, CPP was provided for a total volume of 58 units CPP over 9 days (approximately CPP 227 ml/kg). The authors proposed that CPP CRI may be a viable alternative to CSA (and HSA) for oncotic support and albumin replacement, but multiple units from different donors may increase the risk of an adverse reaction. Cryopoor plasma is only available in specialized institutions or commercial veterinary blood banks.

### Recommendations for Plasma Products Use in Small Animals

With the decline in use of synthetic colloids and in countries where albumin solutions are either not available or not popular, plasma products might be the only natural colloid alternative. However, the current evidence is not sufficient to enable strong recommendations for the use of plasma products in small animals. Allogenic plasma transfusions seem to have a low risk for adverse effects, although cats seem to be more sensitive than dogs. As an albumin source, the administration of plasma (either FFP or CPP) appears to be relatively safe, although large volumes might be necessary. Fresh frozen plasma contains ~30 g/L (3.0 g/dl) of albumin. If the goal of the plasma transfusion is to achieve adequate albumin supplementation, substantial volume per kilogram is needed, particularly when dealing with ongoing albumin loss [i.e., 22 ml/kg of plasma to raise the albumin concentration by 5 g/L (0.5 g/dl)] (152). Administering such volumes, potentially derived from various donors, should be weighed against the risk of volume overload and other transfusion reactions. The use of CPP rather than FFP, where volume expansion is not required, might prevent volume overload. Because these products are usually administered per unit (or ½ unit), because of the necessity to thaw, dose regimens (in ml/kg) vary depending on patient size. Fresh frozen plasma should not be used as a first-line volume expander unless specifically indicated. This is because it is a valuable and expensive resource that should not be wasted, and it is a potential source of adverse reactions in already debilitated animals. Furthermore, it technically cannot be administered as a first-line volume expander due to the necessity to thaw, which takes 30–45 min (209). However, if other indications for FFP administration (e.g., overt or imminent coagulopathy) are present, FFP can be considered as part of resuscitative efforts. While established dosing regimens for FFP as a volume expander do not exist, the authors recommend similar doses as other natural or synthetic colloids.

## Hemoglobin-Based Oxygen Carrier (HBOC) Solutions

Hemoglobin-based oxygen carrier (HBOC) solutions consist of chemically purified and polymerized bovine hemoglobin (Hb) suspended in a crystalloid carrier fluid. The hemoglobin in those solutions is a colloid with a high molecular weight (up to 250 kDa in some) and a relatively high COP. Several products have been developed over the last few decades, seeking the advantage of a long shelf-life and low infectious risk alternative to pRBC transfusions (210). The safety and efficacy of HBOCs has been evaluated in various species mostly through experimental studies and have yielded conflicting results. In hemorrhagic shock models, HBOCs seem to show promising efficacy in restoring cardiovascular parameters and in some studies parallel the oxygen carrying capacity of pRBCs (211–219). However, they are associated with considerable adverse effects such as systemic vasoconstriction, pulmonary and systemic hypertension, reactive oxygen species formation, and renal and myocardial injury (220, 221). As a consequence, HBOCs are not currently authorized for use in people (222).

The use of HBOCs has been described two small-scale clinical studies in dogs. In a prospective randomized study in 20 dogs with GDV, five times smaller volumes of Oxyglobin (OPK Biotech Netherlands BV) were required to achieve resuscitation end-points as compared with HES 450/0.7 (223). Another prospective randomized study compared the use of Oxyglobin and pRBC in 12 anemic dogs with babesiosis. The authors concluded that Oxyglobin had comparable effects with pRBC, with the exception that dogs treated with pRBC had a quicker return to habitus and appetite (224). In a retrospective study about the clinical use of Oxyglobin in 48 cats, a high risk of life-threatening circulatory overload was found in cats suffering from cardiac disease (225). Oxyglobin was authorized by the European Medicines Agency and Food and Drug Administration for use in anemic dogs in 1999 (212–214). However, it has been withdrawn from the market in most countries. Recently, a new highly purified low molecular weight (65 kDa) bovine blood–derived HBOC (Oxapex; New A Innovation Ltd., Kowloon, Hong Kong) has been authorized for use in anemic dogs in New Zealand and awaiting authorization in Australia, the USA, Canada, and the EU (Table 1). A canine hemorrhagic shock model showed improved hemodynamics, lactate, base excess, and tissue oxygenation after administration of various doses of Oxapex (226). In this study, high doses led to significant increases in pulmonary artery pressures (226). Further studies are required to establish the safety and efficacy of this new product.

## Conclusion

Colloids, both natural and synthetic, were originally used with narrow indications. For instance, the original purpose of synthetic colloids was a cheap, long shelf-life “bridging fluid” to remain in the intravascular space while soldiers wounded on the frontline were transported to the field hospital where further stabilization took place. The original purpose of natural colloids was to replace missing blood components (albumin, clotting factors, fibrinogen) in various patients. In the decades that followed, their indications have expanded far beyond their original purpose, such as perioperative fluid therapy for HES and volume expansion for albumin. The expansion of their indications to potentially higher doses and use over longer periods of time (specifically for HES) may have contributed to more and more severe adverse effects being observed and, in some instances, has led to severe restrictions in their use.

Admittedly, veterinary emergency and critical care medicine does, to some extent, take into account the guidelines adopted in human medicine. However, veterinary medicine cannot directly follow the “attitudes” adopted in human medicine because of species differences, different patient cohorts, the availability and safety of some products, and the need to incorporate the owner's financial considerations into the decision-making process. Therefore, species-specific guidelines should be established.

We are living in an era where fluids are recognized as drugs with indications, contraindications, and adverse effects. Therefore, to ensure rational utilization of colloids in the face of weak and contradicting evidence, the authors recommend prescribing colloids on a case-by-case basis, similarly to prescribing any other drug.

## Author Contributions

K-NA and IDY performed the literature search and wrote the article. All authors contributed to the article and approved the submitted version.

## Conflict of Interest

The authors declare that the research was conducted in the absence of any commercial or financial relationships that could be construed as a potential conflict of interest.

## Abbreviations

AKI: acute kidney injury
COP: colloid osmotic pressure
CRI: constant rate infusion
CSA: canine serum albumin
EG: endothelial glycocalyx
ESL: endothelial surface layer
FFP: fresh frozen plasma
Hb: hemoglobin
HBOC: hemoglobin-based oxygen carrier
HES: hydroxyethyl starch
HSA: human serum albumin
EU: European Union
HES: hydroxyethyl starch
HSA: human serum albumin solutions
kDa: kilodalton
pRBC: packed red blood cell
RCT: randomized clinical trial
RRT: renal replacement therapy.
