# Supplementary material for: Colloids Yes or No? - a “Gretchen Question” Answered
Source: Front Vet Sci. 2021 Jul 2;8:624049. doi: 10.3389/fvets.2021.624049 (PMC8282815; doi:10.3389/fvets.2021.624049)
Supplement: Supplementary file 1 [file Data_Sheet_1.docx]

**Table data sheet 1: Overview of pivotal hydroxyethyl starch-studies in people** (sorted by publication year)

| **Acronym** (year) | **Full name of trial/ study group** | **Title of main publication** | **Colloid and comparator solution** | **Study population** | **Major findings** |
| --- | --- | --- | --- | --- | --- |
| **VISEP**  (2008) | Volume Substitution and Insulin Therapy in Severe Sepsis | Intensive insulin therapy and pentastarch resuscitation in severe sepsis. | 10% HES 200/0.5 *versus* modified LRS | Severe sepsis patients (n=600) | HES was associated with significantly higher rates of 90-day mortality (41% vs. 34%), AKI (35% vs. 23%) and RRT (31% vs. 19%). |
| **FIRST**  (2011,) | Fluids in Resuscitation of Severe Trauma | Resuscitation with hydroxyethyl starch improves renal function and lactate clearance in penetrating trauma in a randomized controlled study: the FIRST trial. | 6% HES 130/0.4 *versus* 0.9% NaCl | Penetrating and blunt trauma patients  (n=115) | no differences in AKI in blunt trauma between HES and saline (20% vs. 14%) and a significantly reduced incidence of AKI in penetrating trauma patients treated with HES compared with saline (0% vs. 16%). |
| **6S**  (2012) | Scandinavian Starch for Severe Sepsis/Septic Shock | [Hydroxyethyl starch 130/0.42 *versus* Ringer's acetate in severe sepsis](http://www.ncbi.nlm.nih.gov/pubmed/22738085). | 6% HES 130/0.42 *versus* Ringer’s Acetate | Severe sepsis patients (n=800) | HES was associated with significantly increased 90-day mortality (51% vs. 43%) and use of RRT (22% vs. 16%). |
| **CHEST**  (2012) | Crystalloid versus Hydroxyethyl Starch Trial | Hydroxyethyl starch or saline for fluid resuscitation in intensive care. | 6% HES 130/0.4 *versus* 0.9% NaCl | Intensive care patients (n=7000) | HES was associated with a significant increase in the use of RRT (18% vs. 17%) |
| **CRYSTMAS** (2012) | Crystalloids Morbidity Associated with severe Sepsis | Assessment of hemodynamic efficacy and safety of 6% hydroxyethyl starch 130/0.4 *versus* 0.9% NaCl fluid replacement in patients with severe sepsis: the CRYSTMAS study. | 6% HES 130/0.4 *versus* 0.9% NaCl | Severe sepsis patients (n=196) | no significant differences between the HES and saline groups in AKI (24.5% vs. 20%), 28-day mortality (31% vs. 25.3%) and 90-day mortality (40% vs. 34%). |
| **CRISTAL**  (2013) | Colloids Versus Crystalloids for the Resuscitation of the Critically Ill | Effects of fluid resuscitation with colloids *versus* crystalloids on mortality in critically ill patients presenting with hypovolemic shock: the CRISTAL randomized trial. | HES, gelatins, dextrans, 4% albumin, 20% albumin *versus* hypertonic saline, 0.9% NaCl, LRS | Hypovolemic shock patients  (n=2857) | No significant difference in 28-day mortality between colloids and crystalloids (25.4% vs. 27%) and need for RRT within the first 28 days (11.3% vs. 11.4%). 90-day mortality was higher in the crystalloids group than in the colloids group (mainly HES) (34.2% vs. 30.7%; P=0.03) |
| **RaFTinG**  (2018) | The Rational Fluid Therapy in Germany study | Fluid therapy and outcome: a prospective observational study in 65 German intensive care units between 2010 and 2011 | Colloids (mainly 6% HES 130/0.4, 6% HES 130/0.42, gelatin and albumin) *versus* crystalloids (not specified) | Post-operative ICU patients  (n=4545) | After full multivariate adjustment (e.g., exclusion of patients with severe sepsis and chronic kidney disease), 6% HES 130/0.4 had no remarkable negative effects on 90-day mortality and was even associated with a reduced risk of RRT and AKI |
| **FLASH**  (2020) | Fluid Loading in Abdominal Surgery: Saline vs Hydroxyethyl Starch trial | Effect of hydroxyethyl starch *versus* saline for volume replacement therapy on death or postoperative complications among high-risk patients undergoing major abdominal surgery | 6% HES 130/0.4 *versus* 0.9% NaCl | Major abdominal surgery  (n=775) | HES *vs.* saline resulted in no significant difference in mortality (36% vs. 32%; RR 1.10 (0.91-1.34) P=.33) and AKI (22% vs. 16%; RR 1.34 (1.00-1.80), P=.30) |

AKI, acute kidney injury; HES, hydroxyethyl starch; RRT, renal replacement therapy; vs., *versus*; RR, relative risk
